# Sensitivity to the Demethylation Inhibitor Difenoconazole Among Baseline Populations of Various *Penicillium* spp. Causing Blue Mold of Apples and Pears

**DOI:** 10.3390/jof11010061

**Published:** 2025-01-15

**Authors:** Madan Pandey, Clayton L. Haskell, Juliette D. Cowell, Achour Amiri

**Affiliations:** 1 Department of Plant Pathology, Tree Fruit Research and Extension Center, Washington State University, 1100 N. Western Ave., Wenatchee, WA 98801, USA; 2 Department of Horticulture, University of Minnesota, 1790 Folwell Ave., St. Paul, MN 55108, USA

**Keywords:** postharvest, *Penicillium* spp., baseline populations, cytochrome P450 enzyme, fludioxonil, dual resistance

## Abstract

Difenoconazole (DIF), a demethylation inhibitor fungicide, was registered in 2016 for the control of postharvest diseases of pome fruits. In this study, 162 isolates from *P. expansum* (*n* = 31) and 13 other “non-*expansum*” *Penicillium* spp., i.e., *P. solitum* (*n* = 52), *P. roqueforti* (*n* = 32), *P. commune* (*n* = 15), *P. paneum* (*n* = 9), *P. psychrosexuale* (*n* = 8), *P. crustosum* (*n* = 5), *P. carneum* (*n* = 3), *P. palitans* (*n* = 2), along with one isolate each of *P. citrinum*, *P. griseofulvum*, *P. raistrickii*, *P. ribium*, and *P. viridicatum*, were collected from multiple packinghouses in the U.S. Pacific Northwest. In vitro sensitivity assays showed similar sensitivities of spores and mycelia across species with the mean EC_50_ values ranging from 0.01 for *P. psychrosexuale* (*n* = 8) to 1.33 μg mL^−1^ for *P. palitans* (*n* = 2), whereas the mean EC_50s_ were 0.03, 0.12, 0.19, and 0.51 μg mL^−1^ for *P. expansum* (*n* = 31), *P. paneum* (*n* = 9), *P. solitum* (*n* = 52), and *P. crustosum* (*n* = 5), respectively. The recommended rate of DIF controlled *P. expansum* and *P. roqueforti* isolates but not all isolates of four other *Penicillium* spp. on Fuji apples after five months at 1.5 °C. The mixture of DIF + fludioxonil (FDL) (Academy^TM^) controlled all the dual-sensitive isolates (DIF^S^FDL^S^) and DIF single-resistant (DIF^R^) isolates among the six species tested but not the FDL^R^ and dual DIF^R^FDL^R^ isolates. Notable polymorphism was detected in the *CYP51* gene of the “non-*expansum*” species with four mutations located at four residues. Although the isolates analyzed in this study had not previously been exposed to DIF, the findings indicate variable sensitivity levels among the *Penicillium* spp.

## 1. Introduction

Nearly 100 fungal species have been documented to infect apples and pears at various stages of production and storage [1]. Among these, 10 species can induce economically unsustainable crop losses during storage [1,2]. Pathogens, such as *Alternaria* spp., *Botrytis cinerea*, *Neofabraea* spp., and *Phacidiopycnis* spp., initially infect fruits in orchards and remain latent until favorable humidity conditions arise during storage. Additional pathogens, such as *Penicillium* spp., *Mucor piriformis*, and *Cladosporium* spp., exhibit greater adaptation to postharvest environments, typically infecting fruits through wounds sustained during harvest and/or storage. A variety of factors, including environmental conditions, genetic predispositions, maturity, host susceptibility, along with pre- and postharvest management practices, can influence the incidence of postharvest diseases. Comprehensive surveys conducted in the U.S. Pacific Northwest (PNW) have identified blue mold and gray mold, caused by *Penicillium* spp. and *Botrytis* spp., respectively, as the two most prevalent postharvest diseases, accounting for up to 70% of total decays [2]. Other diseases, such as speck rot, bull’s eye rot, Alternaria rot, Mucor rot, and yellow rot, occurred with varying frequencies [2].

Blue mold, caused by the *Penicillium* species, represents a prevalent and economically significant postharvest disease affecting several fruit crops globally. In the United States, the incidence of blue mold in conventional apples is estimated to range from 1% to 50% of total decay [3,4]. The genus *Penicillium* is recognized as one of the most diverse groups of fungi, encompassing approximately 983 species [5]. In many regions, *P. expansum* is recognized as the primary causal agent of blue mold in pome fruits; however, numerous other *Penicillium* spp. have also been identified as causative agents of blue mold or are commonly found within the postharvest systems of pome fruits. For instance, seven *Penicillium* spp., namely *P. expansum*, *P. solitum*, *P. commune*, *P. verrucosum*, *P. chrysogenum*, *P. rugulosum*, and *P. digitatum*, have been reported on the surfaces of apple fruit both before and after harvest, as well as in the atmosphere of orchards and storage rooms in France [6]. Furthermore, eight *Penicillium* species, i.e., *P. expansum*, *P. commune*, *P. solitum*, *P. aurantiogriseum*, *P. roqueforti*, *P. verrucosum*, *P. glabrum*, and *P. rugulosum*, were recovered from dump tank flotation waters in pear and apple packinghouses in the mid-Columbia region of the PNW [7]. In 2016, five *Penicillium* spp., specifically *P. glabrum*, *P. chrysogenum*, *P. crustosum*, *P. brevicompactum*, and *P. expansum*, were recovered from pear shipments to the United Kingdom originating from South Africa [8,9]. Additionally, various *Penicillium* spp. have been reported on pome fruits in different regions, i.e., *P. solitum* in Uruguay and Serbia [10,11], *P. crustosum* in Serbia, British Columbia, and Korea [11,12,13], *P. italicum* in Korea [13], *P. carneum* in Pennsylvania [14], *P. griseofulvum* in Italy [15], and *P. brevicompactum* in British Columbia [12].

In addition to sanitation measures, postharvest fungicides represent the most effective strategy for the control of postharvest diseases, such as blue mold, in pome fruit. Presently, there are five fungicides approved for postharvest application on pome fruits in the USA and the PNW. Thiabendazole (TBZ), a benzimidazole fungicide registered in the late 1960s, continues to be utilized by packers in combination or alternation with fludioxonil (FDL) and pyrimethanil (PYR), which were registered in 2004. Resistance to TBZ, PYR, and FDL in *P. expansum* isolates has already emerged in the PNW and other growing regions [16,17,18,19]. Recently, high levels of resistance to PYR and FDL have also been documented in 13 *Penicillium* species aside from *P. expansum* [20]. In 2016, the demethylation inhibitor (DMI) fungicide difenoconazole (DIF) was combined with fludioxonil and registered as Academy^TM^ (Syngenta Crop Protection) for the management of postharvest diseases affecting pome fruits. Difenoconazole, marketed as Inspire Super (Syngenta Crop Protection), is also sprayed in orchards to combat apple powdery mildew and other preharvest diseases. Difenoconazole, whether applied alone or in conjunction with other fungicides, has demonstrated efficacy against various pathogens, including *P. expansum* [21,22], *Venturia inaequalis* [23], *Podosphaera leucotricha* [24], *Alternaria alternata* [25], and *Colletotrichum fructicola* [26]. DMI fungicides are categorized as medium risk for resistance development by the Fungicide Resistance Action Committee (FRAC). The DMIs primarily target the 14α- demethylase enzyme, which is encoded by the *CYP51* gene and is essential for the biosynthesis of ergosterol [27]. Mutations in the *CYP51* gene have been documented to confer resistance to DMIs in several fungal pathogens [21,26,28]. However, other mechanisms, other than point mutations, have also been reported to confer resistance to several DMIs [21,23,29,30,31,32,33]. While resistance to DIF has not yet been reported in *Penicillium* species in commercial packinghouses, significant resistance to FDL, the other active ingredient in Academy^TM^, was documented in numerous *Penicillium* species from the PNW [20]. Consequently, it is imperative to evaluate the sensitivity of *Penicillium* species to DIF to ensure the continued effectiveness of Academy.

The baseline sensitivity of *P. expansum* isolates from the Pacific Northwest (PNW) [21] and that of *P. expansum* along with a few other *Penicillium* species from the mid-Atlantic region [22] has been examined. A negligible reduction in sensitivity to DIF was reported among a limited number of “non-*expansum*” *Penicillium* isolates from Pennsylvania or of unknown origin [22]. To assess the potential risk of resistance development to DIF in *P. expansum*, laboratory-selected mutants were tested and were found to be uncontrolled by the label rate of DIF on detached apples [21]. The DIF-resistant mutants exhibited significant overexpression of the *CYP51* gene, as well as a single Y126F substitution in the *CYP51* gene [21]. The sensitivity of numerous other “non-*expansum*” *Penicillium* spp., which constitute up to 25% of the *Penicillium* population in the PNW, remains to be evaluated. The widespread resistance to PYR and FDL [20] necessitates further investigation into the sensitivity of various *Penicillium* spp. to DIF to establish the risks of resistance development in these species, thereby preventing potential control failures. Consequently, this study was conducted to (i) assess the sensitivity of baseline populations of 14 *Penicillium* spp. to DIF in vitro, (ii) evaluate the efficacy of DIF against major *Penicillium* spp. on apples, and (iii) investigate specific target gene mutations in the *CYP51* gene that may potentially confer resistance to DIF.

## 2. Materials and Methods

### 2.1. Collection and Identification of Penicillium spp. Isolates

From 2016 to 2018, multiple packinghouses in the states of Washington and Oregon were surveyed for postharvest decays, resulting in the collection of 162 *Penicillium* spp. isolates from decayed apples and pears (Table S1). The isolates were single-spored, preserved in 20% glycerol at −80 °C, and revived as needed on potato dextrose agar (PDA) at 20 °C for 7 days prior to each experiment. To accurately identify the species of each isolate, four DNA regions, i.e., the internal transcribed spacer (ITS) rDNA, *β-tubulin* (β-Tub), Calmodulin (CaM), and RNA polymerase II (RPB2) were analyzed as described previously [34]. Genomic DNA was extracted from 7-day-old mycelia and conidia scraped from the surface of PDA plates using a phenol-chloroform method [35]. Polymerase Chain Reactions (PCR) were performed as described previously [20,34], and the purified PCR products were subsequently sequenced at Retrogen (San Diego, CA, USA). The resulting sequences were aligned using Geneious (Auckland, New Zealand) with nucleotide sequences of other *Penicillium* spp. available in GenBank and top hits with percent identity values > 98% were considered. The sequence alignments revealed 14 different *Penicillium* species i.e., *P. solitum* (*n* = 52), *P. roqueforti* (*n* = 32), *P. expansum* (*n* = 31), *P. commune* (*n* = 15), *P. paneum* (*n* = 9), *P. psychrosexuale* (*n* = 8), *P. crustosum* (*n* = 5), *P. carneum* (*n* = 3), *P. palitans* (*n* = 2), alongside with one isolate each of *P. citrinum*, *P. griseofulvum*, *P. raistrickii*, *P. ribium*, and *P. viridicatum*.

### 2.2. In Vitro Fungicide Sensitivity Assays

Sensitivity to difenoconazole (Thesis, 32.8% a.i., Syngenta Crop Protection, Greensboro, NC, USA) among the 162 *Penicillium* spp. isolates was evaluated using spore germination and mycelial growth inhibition assays on malt extract agar [MEA; 10 g malt extract and 15 g agar L^−1^ of distilled water (DIW)]. The formulated fungicide was dissolved in DIW to prepare stock solutions of 1000 µg mL^−1^, which were stored at 4 °C for no more than 14 days. To evaluate spore sensitivity, DIF was added to autoclaved and molten MEA at final concentrations of 0, 0.01, 0.05, 0.1, 0.5 µg mL^−1^, selected from preliminary tests. The medium was then poured into 150 mm Petri plates, to which a translucent grid of 10 × 6 squares was affixed beneath to allow simultaneous testing of 60 isolates on each plate. Spore suspensions were prepared by harvesting spores from 7-day-old PDA cultures in DIW containing 0.05% Tween 20. The suspension was then filtered through a double layer of cheesecloth and adjusted to 10^6^ spores mL^−1^ using a hemocytometer. Afterward, a 10 µL droplet of the spore suspension from each isolate was pipetted onto one square of the grid on both control and fungicide-amended plates and incubated for 24 h at 20 °C. Germination and germ tube length were assessed microscopically for 100 spores per treatment. A conidium was considered germinated when its germ tube was at least twice as long as its size.

For mycelial growth inhibition assessment, a total of 88 *Penicillium* spp. isolates (Table S1) were tested for DIF sensitivity. The isolates were cultivated on acidified PDA (APDA, pH = 3) for 10 days at 20 °C to inhibit the production of spores that may interfere with the assay. A 5-mm mycelial plug from the periphery of actively growing APDA colonies was transferred to 90 mm MEA plates supplemented with DIF at the concentrations previously used for spore germination inhibition. The plates were incubated for 5 days at 20 °C, and the colony diameter was measured in two perpendicular directions using a digital electronic caliper (iGaging, San Clemente, CA, USA). For both spore germination and mycelial growth assays, three replicate plates were used for each isolate/fungicide concentration/assay combination, and the experiment was conducted twice. To evaluate cross-sensitivity between the two active ingredients (DIF and FDL) of Academy, the sensitivity of 162 *Penicillium* spp. to FDL (Scholar SC, Syngenta. Crop Protection) was assessed using a spore germination and germ tube inhibition assay, as previously described [20].

### 2.3. Efficacy of Difenoconazole and Academy on Detached Apples

A total of 20 isolates from eight *Penicillium* species (Table S1), exhibiting varying in vitro sensitivity levels to DIF (EC_50_), were selected to evaluate the efficacy of the label rate of DIF in controlling these isolates on detached fruit. Untreated apples (cv. “Fuji”) were harvested at commercial maturity (Firmness = 7.2 kg; Brix = 13.5%) in October 2023 from the WSU Sunrise experimental orchard in Rock Island, WA, and were subsequently stored in a regular atmosphere at 1.5 °C at about 85% relative humidity (RH) until used. The apples underwent a surface sanitation process involving a 2 min immersion in a 1% sodium hypochlorite solution, followed by rinsing with sterile water and allowing them to dry in ambient air. Each apple was punctured around the stem bowl area with a needle (3 mm wide and 4 mm deep) and then immersed for 1 min in a fungicide suspension of Thesis (Syngenta Crop Protection) at a concentration of 0.78 mL L^−1^. In addition to assessing the efficacy of DIF, the efficacies of FDL (Scholar SC) and Academy (DIF + FDL) were evaluated on 16 isolates from six *Penicillium* spp. (Table S1). The apples that were harvested and prepared as described above were then dipped in fungicide suspensions of FDL (Scholar SC) or Academy™ (Syngenta Crop Protection) at the label rate of 1.25 mL L^−1^ for both fungicides. The treated apples were allowed to drain for 1 h at ambient temperature, after which each wound was inoculated with 20 µL of spore suspensions at 10^5^ spores mL^−1^. A randomized complete block design was employed, with four replicates of six apples each for each isolate/fungicide combination. The apples were stored in a regular atmosphere at 1.5 °C and ~85% RH and inspected monthly for five months to determine the incidence and severity of blue mold.

### 2.4. Amplification and Sequencing of the CYP51 Gene in Penicillium spp.

The primer pair CYP51-S5F(TGTGATCAAGCAGCTCGTGT) and CYP51-S1R (CTGCTGTGAAGACGGTTATCT) developed previously [21] was used to amplify an 811 bp fragment of the CYP51 gene from 16 isolates of nine *Penicillium* spp. PCR reactions were conducted in 25 μL volumes and consisted of 13.5 μL EconoTaq PLUS Green 2X Master Mix (EconoTaq PLUS Master Mix, LGC, Biosearch Technologies, Hoddesdon, UK), 1 μL of each 10 μM primer, 0.2 μL bovine serum albumin (BSA), 9.3 μL nuclease-free water, and 1 μL of 20 ng μL^−1^ DNA. The reactions were conducted in BIO-RAD^®^ T100 (T100 Thermal Cycler, Bio-Rad, Hercules, CA, USA) using the following conditions: initial denaturation for 2 min at 95 °C, followed by 30 cycles of 94 °C for 30 s, 55 °C for 1 min, and 72 °C for 1 min, and a final extension at 72 °C for 5 min. The cleaned PCR products were sequenced at Retrogen (San Diego, CA, USA), and the resulting sequences were aligned in Geneious (Auckland, New Zealand) with *CYP51* nucleotide and amino acid sequences of other *Penicillium* spp. from GenBank.

### 2.5. Statistical Analysis

The EC_50_ values of DIF were calculated by plotting the percentage of germination or growth inhibition against the logarithmically transformed concentrations of the fungicide. The incidence of blue mold and the diameters of lesions from the detached fruit assay were analyzed using a one-way ANOVA, with means separated through Tukey’s HSD test at a significance level of *p* < 0.05. Correlations between DIF Log-transformed EC_50_ values for spore germination and mycelial growth inhibition, between Log DIF and Log FDL EC_50_s, and between DIF EC_50_s and blue mold incidence on apples were determined using Pearson correlation analysis. Analyses were carried out using RStudio version 2024.04.0+735 (© 2009–2024 Posit Software, PBC).

## 3. Results

### 3.1. In Vitro Sensitivity to Difenoconazole

**Figure 1:**
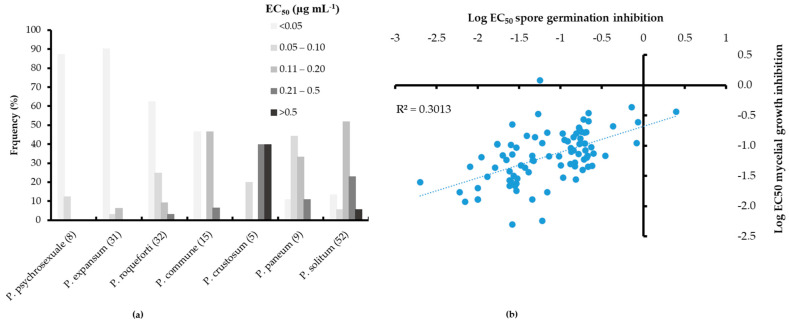
Frequency distribution of the effective concentrations of difenoconazole inhibiting 50% germination and germ tube (EC_50_) among seven *Penicillium* spp. (**a**) and correlation between logarithmically transformed EC_50_ values for spore germination and mycelial growth inhibition (**b**). The numbers in brackets indicate the number of isolates tested for each species.

The mean EC_50_ values for DIF based on spore germination varied significantly among species, ranging from 0.01 µg mL^−1^ in *P. psychrosexuale* (*n* = 8) to 1.33 µg mL^−1^ in *P. palitans* (*n* = 2) (Table 1). The species *P. expansum* (*n* = 31), *P. carneum* (*n* = 3), and *P. roqueforti* (*n* = 32) had mean EC_50_s below 0.1 µg mL^−1^. On the other hand, *P. commune* (*n* = 15), *P. paneum* (*n* = 9), *P. solitum* (*n* = 52), and *P. crustosum* (*n* = 5) exhibited mean EC_50_ values of 0.11, 0.12, 0.19, and 0.51 µg mL^−1^, respectively (Table 1). The EC_50_ value for the single isolate tested for *P. ribium*, *P. griseofulvum*, *P. raistrickii*, *P. citrinum*, and *P. viridicatum* was 0.02, 0.03, 0.11, 0.15, and 0.19 µg mL^−1^, respectively. The lowest variation factors (VFs) were between 5 and 9 and were observed in *P. psychrosexuale, P. carneum*, *P. paneum*, and *P. roqueforti*, whereas the highest VFs of 27, 74, and 138 were, respectively, observed in *P. commune, P. expansum*, and *P. solitum* (Table 1). The EC_50_ values for DIF were left-skewed for *P. psychrosexuale*, *P. expansum,* and *P. roqueforti*, with most isolates exhibiting EC_50s_ < 0.05 µg mL^−1^, whereas the EC_50s_ were normally distributed for *P. paneum* and *P. solitum* (Figure 1a).

A moderate overall positive correlation (R^2^ = 0.301) was observed between the log-transformed EC_50_ values of DIF for mycelial growth and spore germination inhibitions (Figure 1b). The strongest positive correlations were in *P. crustosum* (R^2^ = 0.851, *n* = 3) and *P. commune* (R^2^ = 0.547, *n* = 5). Overall, the mean EC_50_ values for mycelial growth inhibition were lower than those of the spores, ranging between 0.04 µg/mL for *P. psychrosexuale* to 0.22 µg mL^−1^ in *P. palitans*, while the other species had EC_50_ values ranging from 0.05 to 0.18 µg mL^−1^ (Table 1). The VFs based on mycelial growth inhibition were below 20 for most species, except for *P. paneum* (VF = 28) and *P. roqueforti* (VF = 243).

### 3.2. Efficacy of Difenoconazole to Control Penicillium spp. Isolates on Apples

**Figure 2:**
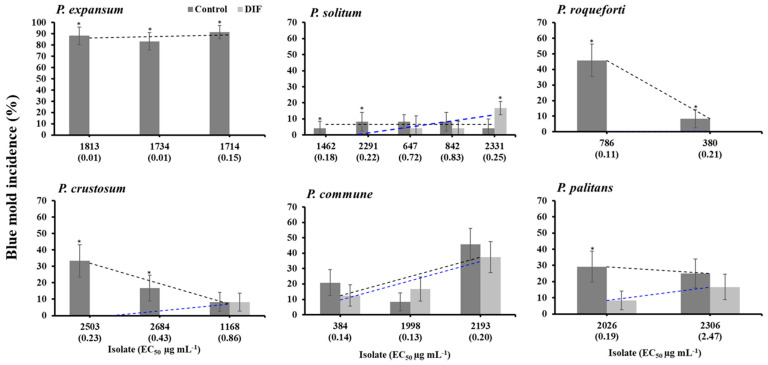
Blue mold incidence after five months of storage at 1.5 °C on untreated (control) and DIF-treated Fuji apples then inoculated with spore suspensions of *Penicillium* spp. isolates with different EC_50_ values shown in brackets. Bars indicate the standard deviations of the means, and an asterisk indicates a significant difference between the incidence in the control and DIF for each isolate based on Tukey’s HSD test at *p* < 0.05. Dashed black and blue lines indicate regression between EC_50_ values and blue mold incidence (%) for each *Penicillium* spp., for the control and DIF treatments, respectively.

**Figure 3:**
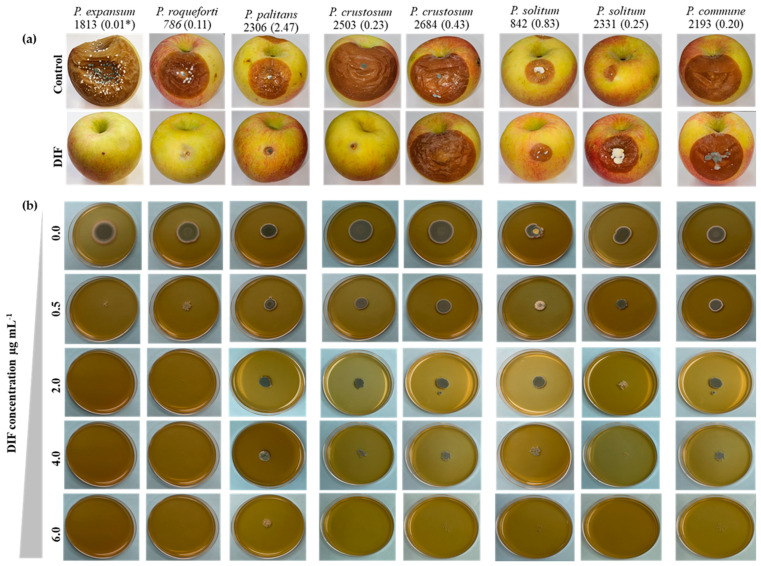
(**a**) Blue mold lesions after five months of storage at 1.5 °C on untreated (control) and DIF-treated Fuji apples then inoculated with spore suspensions (10^5^ spores mL^−1^) of each isolate. An asterisk indicates the EC_50_ value (µg mL^−1^) of each isolate; (**b**) Growth of *Penicillium expansum*, *P. roqueforti*, *P. palitans*, *P. crustosum*, *P. solitum*, and *P. commune* isolates after 7 days at 22 °C on malt extract agar medium supplemented with different DIF concentrations.

After five months of storage at 1.5 °C, the 18 isolates from eight *Penicillium* spp. were pathogenic on untreated “Fuji” apples. The incidence of blue mold ranged from 4.2% in *P. griseofulvum* and *P. raistrickii* to 91.7% in *P. expansum* (Figure 2). The overall blue mold incidence caused by the three *P. expansum* isolates was 86.1% on untreated apples, whereas DIF fully controlled the three isolates tested after five months regardless of their EC_50_ value (Figure 2 and Figure 3a). Similarly, the two *P. roqueforti* isolates with EC_50s_ of 0.11 and 0.21 µg mL^−1^ caused 8.3 to 45.8% blue mold incidence on untreated apples but were fully controlled by DIF (Figure 2 and Figure 3a). However, the label rate of DIF failed to control all isolates of *P. solitum*, *P. commune*, *P. crustosum*, and *P. palitans*. The two *P. palitans* isolates caused 25 to 29.2% blue mold incidence on untreated apples and were not controlled by DIF after five months of storage (Figure 2). DIF controlled *P. crustosum* isolates with EC_50s_ < 0.5 µg mL^−1^ but not isolates 1168 which had an EC_50_ value of 0.86 µg mL^−1^ (Figure 2 and Figure 3a). Three *P. solitum* isolates, 2331, 647, and 842, with receptive EC_50s_ of 0.32, 0.72, 0.83 µg mL^−1^, were not controlled by DIF, resulting in blue mold incidences ranging from 4.2 to 8.3% on untreated apples and from 4.2 to 16.7% on DIF-treated apples (Figure 2 and Figure 3a). The incidence of blue mold caused by *P. commune* isolates ranged from 8.3 to 45.8% on untreated apples, and DIF failed to control all isolates tested regardless of their EC_50s_ ranging from 0.13 to 0.20 µg mL^−1^ (Figure 2 and Figure 3a).

The growth of *P. expansum* and *P. roqueforti* was inhibited on MEA amended with 0.5 µg mL^−1^ after 7 days at 20 °C, while isolates of *P. crustosum*, *P. solitum*, and *P. commune* grew on up to 4.0 µg mL^−1^ DIF (Figure 3b). The lesion size of *P. palitans* isolate 2306 was significantly reduced by DIF (Figure 3a), but the isolate grew on MEA amended with 6.0 µg DIF mL^−1^ (Figure 3b). The size of the blue mold lesions was significantly lower in the “non-*expansum*” isolates which caused lesions with diameters ranging from 0.4 mm to 28.9 mm and from 0.0 to 10.6 mm on untreated and treated apples, respectively (Table S2).

### 3.3. Cross-Sensitivity to Difenoconazole and Fludioxonil Among Penicillium spp.

A moderate positive significant correlation (*r* = 0.427, *p* < 0.001) was observed between the sensitivities of DIF and FDL across all species with more than three isolates (*n* = 155). A strong positive significant correlation was observed between the sensitivities of DIF and FDL in *P. commune* (*r* = 0.946, *p* ≤ 0.0001) and *P. crustosum* (*r* = 0.908, *p* = 0.033). In contrast, a moderate positive significant correlation was observed in *P. expansum* (*r* = 0.578, *p* = 0.0006) (Table 2). Conversely, a significant negative correlation and a weak negative non-significant correlation were observed in *P. roqueforti* (*r* = −0.438, *p* = 0.012) and *P. solitum* (*r* = −0.051, *p* = 0.718), respectively. Furthermore, moderate to strong non-significant correlations were observed in *P. paneum* (*r* = 0.588, *p* = 0.125) and *P. carneum* (*r* = 0.897, *p* = 0.291), whereas a strong positive correlation was observed in *P. psychrosexuale* (*r* = 0.753, *p* = 0.031). The frequency of dual-resistant isolates DIF^R^FDL^R^ was higher in *P. crustosum* (60%), *P. commune* (53.3%), and *P palitans* (50%), followed by *P. solitum* (19.2%) (Table 2). None of the *P. expansum* and *P. roqueforti* isolates exhibited dual resistance to DIF and FDL.

As illustrated in Figure 2 and Table 2, DIF failed to control all isolates except for *P. expansum* and *P. roqueforti*. Moreover, FDL was ineffective against isolates from most *Penicillium* spp. with higher EC_50_ values. The *P. solitum* isolate 2331, *P. crustosum* isolate 2684, *P. palitans* isolate 2026, and *P. commune* isolates 1998 and 2193 were unresponsive to either fungicide. The combination of DIF and FDL (Academy^TM^) successfully controlled all dual-sensitive isolates (DIF^S^FDL^S^) and DIF single-resistant (DIF^R^) isolates among the species evaluated; however, it was ineffective against the FDL single-resistant isolates (FDL^R^) and dual DIF^R^FDL^R^ isolates (Table 2).

### 3.4. Sequencing and Analysis of the CYP51 Gene from Different Penicillium spp. Isolates

A fragment of 811 bp of the *CYP51* gene was amplified from 16 isolates representing nine *Penicillium* species, selected based on their in vitro and in vivo sensitivities to DIF. The amino acid sequences were aligned with reference sequences of several *Penicillium* spp. from GenBank, while the *P. expansum* reference sequence MH507024.1 was used for amino acid annotations. None of the 16 sequenced isolates, irrespective of species or EC_50_ value, exhibited the Y126F mutation (Table 3), which has been previously reported to confer DIF resistance in *P. expansum* laboratory mutants. Two concurrent mutations were identified at codon 92 (V92I) and codon 169 (D169N) in two *P. solitum* isolates and one *P. commune* isolate, which had EC_50_ values of 0.18, 0.72 µg mL^−1^, respectively, that were not effectively controlled by DIF (Figure 2 and Figure 3). In addition to these two substitutions, an I140V mutation was detected at residue 140 of *CYP51* in *P. crustosum* isolates 1168 and 2684, which were not controlled and controlled, respectively, by DIF on detached apples, as well as in *P. carneum* isolate 972. A serine-to-asparagine mutation was observed in *P. paneum*, *P. roqueforti*, and *P. carneum* isolates, which did not exhibit mutations at any of the other three codons. Finally, the aspartic acid residue at codon 169 was mutated to asparagine in nearly all “non-*expansum*” isolates (Table 3). The latter mutation was found to occur concurrently with or without other mutations at additional *CYP51* residues.

## 4. Discussion

Since its registration in 2016, the demethylation inhibitor (FRAC 3) fungicide DIF has not been extensively utilized in the PNW relative to other postharvest fungicides. This limited application can be primarily attributed to the absence of a fog formulation, which is the predominant method employed by pome fruit packers in the region for the application of postharvest fungicides. The low input of DIF in packinghouses has not selected for resistance yet in the major blue mold-causing species *P. expansum*, consistent with prior findings regarding larger populations in the PNW [21] and Mid-Atlantic [22] regions. This study presents a comprehensive evaluation of the sensitivity of various *Penicillium* species to DIF and assesses its efficacy in controlling blue mold on detached apples. The results provide critical insights into species-specific responses, cross-sensitivity to fludioxonil (FDL), and the potential genetic mechanisms underlying the observed variability in sensitivity levels among species.

The *in vitro* sensitivity assays revealed significant variability in the sensitivity of *Penicillium* species to DIF. The mean EC_50_ values for spore germination and mycelial growth inhibition ranged from 0.01 μg mL^−1^ in *P. psychrosexuale* to 1.33 μg mL^−1^ in *P. palitans*. Notably, *P. expansum*, the predominant causal agent of blue mold, exhibited a comparatively low mean EC_50_ value of 0.03 μg mL^−1^. This sensitivity aligns with previous studies that reported minimal resistance in baseline populations of *P. expansum* to DIF [21,22]. In contrast, the broader spectrum of “non-*expansum*” *Penicillium* species displayed a wider range of sensitivities, with higher EC_50_ values recorded for *P. solitum* and *P. crustosum*. Relative to other fungal pathogens, the EC_50_ values observed in this study fall within the typical range documented for DIF sensitivity. For instance, *Venturia inaequalis* and *Alternaria alternata*, two significant plant pathogens, exhibited EC_50_ values for DIF ranging from 0.01 to 0.5 μg mL^−1^ and 0.03 to 0.25 μg mL^−1^, respectively [23,25]. Like *P. expansum*, these pathogens demonstrated high sensitivity to DIF, establishing it as an effective control agent. However, EC_50_ values for other fungi, such as *Monilinia* and *Colletotrichum fructicola*, can reach up to 1.0 μg mL^−1^, indicating reduced sensitivity in comparison with the more susceptible species [26,36]. The elevated EC_50_ values observed in *P. solitum* and *P. crustosum* are more consistent with these less sensitive fungal species, underscoring the necessity for alternative management strategies for these “non-*expansum*” species. The observed variation factors (VFs) within species, particularly in *P. solitum* (VF = 138) and *P. expansum* (VF = 74), highlight a broad spectrum of sensitivity to DIF. This heterogeneity may arise from underlying genetic polymorphisms, environmental pressures, or variations in intrinsic metabolic pathways. Similar variability has been documented in other fungal species, such as *V. inaequalis*, which exhibited VFs of up to 100 for DMI fungicides due to differences in *CYP51* gene expression and mutations [23]. The VFs reported in this study indicate that resistance management strategies should consider intra-species variability, particularly in “non-*expansum*” species, where elevated VFs may complicate predictions regarding fungicide efficacy. Finally, the significant positive correlation (R² = 0.301) between EC_50_ values for spore germination and mycelial growth inhibition is consistent with findings reported in *A. alternata* [25]. These correlations suggest that DIF sensitivity is generally consistent across different fungal growth stages, thereby validating the reliability of both assays in predicting fungicide efficacy across various developmental stages, which is essential for assessing the field-level effectiveness of DIF.

Difenoconazole (Thesis^TM^) at the label rate effectively controlled blue mold caused by *P. expansum* and *P. roqueforti* after five months of storage at 1.5 °C. The high efficacy of DIF against these species, specifically *P. expansum*, aligns with the observed low EC_50_ values *in vitro*, supporting its utility as a postharvest treatment. However, DIF did not control all the isolates of *P. solitum*, *P. crustosum*, *P. commune*, and *P. palitans*. This discrepancy underscores a species-specific risk of resistance development which should be considered when designing fungicide application programs. Previous studies have similarly reported effective control of *V. inaequalis* and *A. alternata* by DIF, while on the other hand, its efficacy diminishes against less sensitive fungi, such as *C. fructicola* [23,25]. The combination of DIF and FDL (Academy™) provided broader control, effectively managing dual-sensitive (DIF^S^FDL^S^) and single DIF-resistant (DIF^R^) isolates. However, it could not control isolates resistant to both fungicides (DIF^R^FDL^R^), emphasizing the need for monitoring resistance and rotating fungicides with different modes of action. These findings are consistent with prior observations that fungicide mixtures can enhance control efficacy but may not overcome dual resistance, as reported for *V. inaequalis* and Colletotrichum gloeosporioides [23,37]. Compared with other fungal pathogens, the efficacy of Academy™ is similar to its performance against *C. fructicola* and *A. alternata*, where mixtures of DMI fungicides and other active ingredients have been shown to enhance control [25,36]. Interestingly, no additive or synergistic effects were observed between DIF and FDL in controlling resistant *Penicillium* isolates. This observation is consistent with findings in other pathogens, such as *Botrytis cinerea* and *Colletotrichum gloeosporioides*, where mixtures of DMI fungicides and other active ingredients showed limited synergy and were ineffective against highly resistant isolates [36,37]. The absence of control over FDL-resistant isolates by Academy™ suggests that fludioxonil is the most effective component in the mixture against *Penicillium* spp. Furthermore, the elevated levels of FDL resistance documented recently in *Penicillium* spp. [20] pose a potential risk to the efficacy of Academy™ in managing blue mold caused by “non-*expansum*” species. The lack of synergy in these combinations underscores the potential for cross-resistance and emphasizes the importance of incorporating non-chemical strategies, including sanitation and cultural practices, to reduce fungal inoculum in storage environments into disease management.

Sequencing of the *CYP51* gene has revealed considerable genetic polymorphism among “non-*expansum*” *Penicillium* species, with mutations identified at codons 92 (V92I), 140 (I140V), and 169 (D169N). These mutations were present in isolates exhibiting varying EC_50_ values; however, their role in conferring resistance to difenoconazole (DIF) remains ambiguous. Notably, none of the isolates contained the Y126F mutation previously associated with DIF resistance in laboratory-selected *P. expansum* mutants [21]. The absence of this mutation in baseline populations indicates that resistance mechanisms may differ between *P. expansum* and “non-*expansum*” species. The frequent occurrence of the D169N mutation in the “non-*expansum”* species, yet its absence in *P.* expansum, raises questions regarding its functional significance, implying it may represent a natural polymorphism rather than a mutation associated with resistance. Recently, no fewer than seven substitutions, i.e., an I133V equivalent to I140V detected in *Penicillium* spp. from the PNW, were found in the *CYP51A* gene of *Pyrenophora teres* f. *teres* isolates with different sensitivity levels to DMI in Northern Europe [28]. The concurrent presence of multiple mutations in certain isolates indicates that a combination of genetic alterations may contribute to the observed variability in sensitivity. Beyond mutations in *CYP51*, additional mechanisms, such as the overexpression of efflux pumps, alterations in sterol biosynthesis pathways, and general stress response mechanisms, have been recognized as factors contributing to resistance in other fungal species [29,30]. For instance, the overexpression of ATP-binding cassette (ABC) transporters has been correlated with DMI resistance in *P. digitatum* and *V. inaequalis* [23,31]. Similarly, changes in the functionality of the sterol 14α-demethylase enzyme, resulting from non-synonymous mutations in the *CYP51* gene, have been reported in *Parastagonospora nodorum* and *Mycosphaerella graminicola* as critical resistance mechanisms [32,33]. These findings highlight the intricate interplay between genetic mutations and other resistance mechanisms, indicating that effective management of resistance to DIF will necessitate a multifaceted strategy that addresses both target-site alterations and non-target-site resistance.

## 5. Conclusions

The findings underscore the necessity for proactive resistance management strategies to sustain the efficacy of DIF and other postharvest fungicides. The variability in sensitivity among *Penicillium* species, alongside the documented resistance to FDL, emphasizes the importance of implementing integrated approaches. Regular monitoring of fungicide sensitivity in baseline populations is essential for detecting shifts in resistance and guiding fungicide application decisions. Furthermore, the alternation of fungicides with distinct modes of action can reduce selection pressure and delay the emergence of resistance. The use of mixtures such as DIF + FDL (Academy™) can enhance control efficacy and reduce the development of resistance; however, their effectiveness against dual-resistant isolates remains constrained. Additionally, in light of the reported ineffectiveness of several postharvest fungicides against these species [20], improved sanitation and storage practices will be crucial for decreasing fungal inoculum and minimizing decay during long-term storage.

## Figures and Tables

**Table 1:** Mean and range of effective concentration inhibiting 50% germination or growth (EC_50_) of difenoconazole and variation factors among *Penicillium* spp. isolates tested in this study.

	Mean and Range of EC_50_ (µg mL^−1^) and Variation Factors for Difenoconazole							
*Penicillium*	Spore Germination Inhibition				Mycelial Growth Inhibition			
Species	N ^1^	Mean	Range	VF ^2^	N ^1^	Mean	Range EC_50_	VF
*P. solitum*	52	0.19	0.006–0.83	138	29	0.11	0.03–0.44	16
*P. roqueforti*	32	0.05	0.022–0.21	9	15	0.15	0.01–1.21	243
*P. expansum*	31	0.03	0.002–0.15	74	16	0.05	0.01–0.14	11
*P. commune*	15	0.11	0.008–0.21	27	5	0.09	0.05–0.14	3
*P. paneum*	9	0.12	0.037–0.22	6	6	0.12	0.05–0.16	28
*P. psychrosexuale*	8	0.01	0.002–0.01	5	4	0.04	0.01– 0.09	7
*P. crustosum*	5	0.51	0.076–0.95	12	3	0.18	0.10–0.25	3
*P. carneum*	3	0.03	0.007–0.05	6	3	0.08	0.01–0.23	19
*P. palitans*	2	1.33	0.190–2.47	13	2	0.22	0.06–0.37	6

^1^ Number of isolates tested for each species. ^2^ Variation factors (VFs) were calculated by dividing the highest EC_50_ values by the lowest EC_50_ value for each species.

**Table 2:** Correlation between the EC_50_ values of difenoconazole and fludioxonil, frequency of dual-resistant isolates to both fungicides, and their efficacy on detached apples.

		n (%)				Efficacy of the Fungicide on Detached Apples ^4^					
*Penicillium*		Dual-Resistant	Correlation Between DIF and FDL ^3^				Difenoconazole		Fludioxonil		Academy
Species	N ^1^	DIF^R^FDL^R 2^	R^2^	r	*P*	Isolate	EC_50_ (µg/mL)	Incidence	EC_50_ (µg/mL)	Incidence	Incidence
*P. solitum*	52	10 (19.2)	0.003	−0.051	0.718	1462	0.18	0.0 ± 0.0	1.40	29.2 ± 7.3	18.9 ± 3.5
						2331	0.25	16.7 ± 7.8	202.7	4.2 ± 4.2	43.8 ± 9.1
						647	0.72	4.2 ± 4.2	0.03	0.0 ± 0.0	0.0 ± 0.0
*P. expansum*	31	0 (0.0)	0.334	0.578	0.0006	1734	0.01	0.0 ± 0.0	0.04	0.0 ± 0.0	0.0 ± 0.0
						1714	0.15	0.0 ± 0.0	0.34	0.0 ± 0.0	0.0 ± 0.0
						1813	0.01	0.0 ± 0.0	0.65	20.8 ± 8.1	12.5 ± 1.7
*P. commune*	15	8 (53.3)	0.896	0.946	<0.0001	384	0.05	12.5 ± 6.9	1.0	0.0 ± 0.0	0.0 ± 0.0
						1998	0.13	16.7 ± 7.8	5.0	4.1 ± 5.0	15.0 ± 2.4
						2193	0.20	37.5 ± 10.1	>500	12.5 ± 6.9	6.3 ± 4.9
*P. crustosum*	5	3 (60.0)	0.824	0.908	0.033	2503	0.23	0.0 ± 0.0	0.04	0.0 ± 0.0	0.0 ± 0.0
						2684	0.43	8.3 ± 5.8	1.76	4.2 ± 6.6	9.8 ± 1.8
						1168	0.86	0.0 ± 0.0	>500	12.5 ± 5.9	15.0 ± 5.7
*P. roqueforti*	32	0 (0.0)	0.192	−0.438	0.012	786	0.11	0.0 ± 0.0	21.70	8.3 ± 8.4	17.5 ± 4.3
						380	0.21	0.0 ± 0.0	0.49	0.0 ± 0.0	0.0 ± 0.0
*P. palitans*	2	1 (50.0)	1.000	–	–	2026	0.19	8.3 ± 5.8	>500	29.2 ± 9.5	18.8 ± 3.7
						2306	2.47	16.7 ± 7.8	1.00	0.0 ± 0.0	0.0 ± 0.0

^1^ Number of isolates tested for each species for both fungicides. ^2^ Number and frequency (%) of isolates characterized as dual-resistant to DIF and FDL (DIF^R^FDL^R^). ^3^ Spearman coefficient correlation (*r*) and significance (*p*) between EC_50_ values of DIF and FDL based on spore germination inhibition assay. ^4^ Mean ± standard deviations of blue mold incidence after five months of storage at 1.5 °C.

**Table 3:** Mutations detected in the *CYP51* gene of *Penicillium* spp. isolates with different in vitro and in vivo sensitivity levels.

*Penicillium*		EC_50_ for DIF (µg/mL)		Incidence	Amino Acid at Codon				
Species	Isolate	Spores	Mycelial	on Apples (%) ^1^	V92 ^2^	Y126	I140	S161	D169
*P. expansum*	1813	0.01	0.03	0.00	V	Y	I	S	D
	1734	0.01	0.02	0.00	V	Y	I	S	D
	1714	0.15	0.05	0.00	V	Y	I	S	D
*P. solitum*	1462	0.18	0.17	0.00	**I**	Y	I	S	**N**
	647	0.72	0.44	4.20	**I**	Y	I	S	**N**
*P. paneum*	3114	0.07	0.17	–	V	Y	I	**N**	**N**
	794	0.22	0.35	–	V	Y	I	**N**	**N**
*P. roqueforti*	786	0.11	0.03	0.00	V	Y	I	S	D
	898	0.02	0.04	–	V	Y	I	**N**	**N**
*P. crustosum*	1168	0.86	0.25	0.00	**I**	Y	**V**	S	**N**
	2684	0.43	0.21	8.30	**I**	Y	**V**	S	**N**
*P. carneum*	2330	0.05	0.01	–	V	Y	I	**N**	**N**
	972	0.03	0.23	–	V	Y	**V**	**N**	**N**
*P. commune*	384	0.05	0.14	12.50	**I**	Y	I	S	**N**
*P. psychrosexuale*	2228	0.01	0.05	–	V	Y	I	**N**	**N**
*P. viridicatum*	792	0.19	0.28	–	V	Y	I	**N**	**N**

^1^ Mean blue mold incidence after five months of storage at 1.5 °C in a regular atmosphere on Fuji apples treated with difenoconazole (DIF) at the label rate. - indicates isolate not tested or not pathogenic on fruit. ^2^ Bold amino acids indicate a mutation compared with the amino acid shown at the top of the table from the wild-type *P. expansum* reference (GenBank accession # MH507024.1).

## Data Availability

The original contributions presented in this study are included in the article and Supplementary Materials. Further inquiries can be directed to the corresponding authors.

## Supplementary Materials

The following supporting information can be downloaded at https://www.mdpi.com/article/10.3390/jof11010061/s1, Table S1: List and origin of *Penicillium* spp. isolates used in this study; Table S2: Severity (lesion diameter) of blue mold caused by *Penicillium* spp. with different sensitivity level to difenoconazole after 5 months of storage.
